# Frequencies of gastrointestinal parasites among students of primary school in Al Kalakla Locality, Khartoum State, Sudan: a cross-sectional study

**DOI:** 10.12688/f1000research.20610.3

**Published:** 2020-09-07

**Authors:** Hala Abdalazim Hassan, Ahmed Bakheet Abd Alla, Tayseer Elamin Mohamed Elfaki, Mohammed Baha Eldin Ahmed Saad

**Affiliations:** 1 Department of Parasitology and Medical Entomology, College of Medical Laboratory Science, Sudan University of Science and Technology, Khartoum, Khartoum, 11111, Sudan; 2 Department of Parasitology and Medical Entomology, College of Medical Laboratory Science, Omdurman Ahlia University, Khartoum, Khartoum, 11111, Sudan

**Keywords:** Al-kalakla, E. histolytica, H. nana, formal ether concentration technique, intestinal parasite, frequencies, Khartoum.

## Abstract

**Background:** Intestinal parasite spread in tropical countries is especially common among primary school students. This study aimed to determine the frequencies of the intestinal parasite by different techniques among school students in Alkalakla locality, Khartoum state.

**Methods:** This study was conducted in school students in Al-kalakla locality in Khartoum state from period between 20th December 2016 to 5th May 2017. Stool samples were collected from 134 randomly selected students, of whom 67 were males and 67 were females.  All samples were examined using the wet preparation technique, formal ether concentration technique and saturated sugar floatation technique.

**Results:** The frequency of intestinal parasites was 35.5% overall in the students examined; females were more affected than males (38.8% and 32.8%, respectively). The more affected age groups were 12-14 years followed by 9-11 and 6-8 years old (53.8%, 36.3% and 26.4% respectively). The least frequent intestinal parasite was *Taenia *spp. (1.5%) followed by *Giardia lamblia* (3.7%), *Schistosoma mansoni* and *Ascaris lumbricoides* (5.2% each), *Entamoeba coli* (7.5%), *Hymenolepis nana* (10.4%), and *Entamoeba histolytica* (16.4%). In total, 20.9% were infected with single parasite while 14.9% were infected with more than one parasite. The frequency of parasite by formal ether concentration method was 35.8 %, by wet preparation method was 17.9 % and by the saturated sugar flotation method was 16.4%.

**Conclusion: **Our data showed that intestinal parasites were common in school students; however, females were more affected than males and the 12-14-years age group was the most affected age group.  The formal ether concentration method was the best method for detecting of intestinal parasite.

## Introduction

Intestinal parasites, particularly in tropical and subtropical areas, are a significant health issue ^1^. Approximately 3.5 billion individuals are estimated to be impacted in developing nations and 450 million are sick as a consequence of these diseases, the majority being children ^2^. Approximately one-quarter of the world's population is infected with intestinal parasites and about 80% of all deaths in developing nations are caused annually by infectious and parasitic illnesses ^3^. There is a powerful correlation between the elevated incidence of these diseases and poverty, bad environmental health and insufficient health facilities ^3^. Also involved is poor personal hygiene, an unsafe water supply and an absence of health education ^4^. The transmission of intestinal parasites is based on characteristics of the parasite, actions of the individual and ecological and biological factors ^5^. Transmission occurs by ingestion of contaminated fecal food or water, by hands contaminated with fecal matter coming into contact with the mouth or by skin penetration by larval stage of the parasite following direct contact with contaminated fecal soil ^6^. Children of school-age are especially prone to symptoms, sometimes carrying a greater burden of parasites than adults ^7^. Diagnosis is routinely performed using a microscope, with fecal samples prepared for microscopy by direct wet mounting or concentration methods. Although direct wet mounting has low sensitivity ^8, 9^, it is still used in low- and middle-income countries ^9^. There are many methods to concentrate the stages of intestinal parasites; cysts, eggs and larvae can be analyzed as specimens using techniques such as formal ether sedimentation and flotation ^10^. These techniques are better than direct wet mounting since they identify more parasites ^11^.

## Methods

### Study area and design

This study is a cross-sectional study conducted in the locality of Al-Kalakla in the state of West Khartoum. This study was carried out between December 2016 and May 2017.

### Study population

The study population was children at a primary school (Saeed primery boys school, Ebaid Khatiem primery boys school, the martyr Abdulsalam primary girls school and Ebaid Khatiem primery girls school), between the ages of 6 and 14. The purpose of the study was explained to the guardians, students and head of school and a total of 134 participants agreed to participate in this study, they were selected randomly. The children were split into three age groups (6–8, 9–11, 12–14 years) and the same number of male and female participants was selected (n=67 of each). A labeled, large-mouth stool container was given to each selected student for collection of fecal samples. Samples were collected during the school day, all samples were subjected to examine by direct smear examination, formal ether concentration technique and Saturated sugar flotation technique.

### Direct smear examination

Wet preparation was achieved by blending a small part of the stool sample taken with a wooden applicator with a drop of normal saline on a slide. The sample was enclosed with a cover slip and systematically examined under a microscope using a 10X lens and elevated magnification 40X lens for further detail observation, after searching for three slides without detecting intestinal parasite, it was considered a negative result.

### Formal ether concentration technique

Approximately 1 g of feces from separate areas of the specimen was gathered and emulsified in glass beaker in 5 ml of formal saline. A further 5 ml were added and mixed from the same solution. The resulting suspension was strained through a strainer. The filtered sample was poured back into a centrifuge tube and an equal quantity of ether was added. The tube was shaken by hand for 1 min and then centrifuged at 2000 rpm for 5 minutes. The upper three layers were removed and the sediment was transported to a slide covered with a cover slip and examined at 10X and 40X magnification under a light microscope.

### Saturated sugar flotation technique

Approximately 100 ml of saturated sugar solution (dextrose) was put into a glass measuring cup and approximately 1 g of stool sample was added. The fecal sample was blended with the saturated sugar solution for flotation. The fecal debris was filtered into another cup and the remaining fluid was drawn out. The preparation that was filtered was poured into a glass test tube. To the top of the tube was added fecal flotation solution. A cover slip was placed on the top of the tube. The pipe remained unchanged for 15 to 30 minutes and the covering glass was closely put on a slide and microscopically examined ^12^.

### Identification of parasites

*E. histolytica, G. lamblia* and *Entamoeba coli* were identified by presence of a cyst or trophozoite in the stool. *Taenia* spp., *Schistosoma mansoni*, *Ascaris lumbricoides*, and *Hymenolepis nana* were identified by presence of eggs of each helminth in the stool.

### Data analysis

Data were analyzed using Statistical Package for Social Sciences (version 16). Using Chi square test, the significant obtained when P < 0.05. Data were presented in tables.

The sensitivity and specificity of each technique was calculated using the two formulae below:

$$\text{Sensitivity}\;\text{=}\;\frac{\text{positive}\;\text{cases}\;\text{of}\;\text{tested}\;\text{technique}}{\text{positive}\;\text{cases}\;\text{of}\;\text{reference}\;\text{technique}}\text{×}100$$

$$\text{Specifity}\;\text{=}\;\frac{\text{negative}\;\text{cases}\;\text{of}\;\text{tested}\;\text{technique}}{\text{negative}\;\text{cases}\;\text{of}\;\text{reference}\;\text{technique}}\text{×}100$$

### Ethical consideration

Ethical clearance for this study was obtained from Committee of medical laboratory science, Sudan University of Science and Technology, ethical approval number (MLS – IEC – 08 – 16). Written informed consent for participation and publication of the data was obtained from the guardians of all participants included in this study.

## Results

### Overall occurence of intestinal parasites

Analysis showed that 48 of the 134 stool samples collected from participants were positive for gastrointestinal parasites in Al-kalakla locality, Khartoum state. This was an overall prevalence rate of 35.8%. Infection statuses of all participants, alongside the methods used to identify infection, are available as *Underlying data* ^13^.

### Occurence of intestinal parasite by variables

The research disclosed that in females the occurrence of gastrointestinal parasites was 38.8% while in males it was 32.8%. The difference in gender rates was found to be statistically insignificant (P=0.471; Table 1). Findings showed that age groups 6–8, 9–11 and 12–14 years had parasite prevalence rates of 26.4%, 36.3% and 53.8%, respectively. These variations in rates at P = 0.057 ( Table 2) were statistically insignificant. The prevalence of different parasites was found as follows: *E. histolytica* (16.4%), *H. nana* (10.4%), *E. coli* (7.5%), *A. lumbricoides* (5.2%), *S. mansoni* (5.2%), *G. lamblia* (3.7%) and *Taenia* spp. (1.5%) ( Table 3). The results showed that 28 (20.9%) were infected with single parasite and that there were 20 infected with more than one parasite (14.9%) ( Table 4).

**Table 1.** Prevalence of intestinal parasites among gender.

Gender	Number examined	Number positive	Prevalence (%)
Males	67	22	32.8
Females	67	26	38.8
Total	134	48	35.8

P = 0.471

**Table 2.** Prevalence of intestinal parasite according to age group.

Age group (years)	Number examined	Number positive	Prevalence (%)
6–8	53	14	26.4
9–11	55	20	36.3
12–14	26	14	53.8

P = 0.057

**Table 3.** Prevalence of intestinal parasite according to species.

Parasite	Number examined	Number positive	Prevalence (%)
*E. histolytica*	134	22	16.4
*H. nana*	134	14	10.4
*E. coli*	134	10	7.5
*A. lumbricoides*	134	7	5.2
*S. mansonia*	134	7	5.2
*G. lamblia*	134	5	3.7
*Taenia* spp.	134	2	1.5

**Table 4.** Prevalence of intestinal parasite according to type of infection.

Type of infection	Number examined	Number positive	Prevalence (%)
Single	134	28	20.9
Mixed	134	20	14.9

### Correlation between parasitological techniques

The frequency of gastrointestinal parasites by various parasitological methods was as follows: 35.8% by formal concentration method, 17.9% by the wet preparation method and 16.4% by the saturated sugar flotation method. A comparison between the detection rate of each method found that detection rates were significantly different (P = 0.000; Table 5).

**Table 5.** Correlation between parasitological techniques.

Parasitological technique	Number examined	Number positive	Prevalence
Wet preparation technique	134	24	17.9%
Formal ether concentration technique	134	48	35.8%
Saturated sugar flotation	134	22	16.4%

P = 0.000

### Sensitivity and specificity of techniques compared with formal ether

Assuming that the formal ether concentration technique was the gold standard, the sensitivity and specificity of the wet preparation technique was 50% and 100% respectively, and the sensitivity and specificity of the saturated sugar flotation technique was 45% and 100%, respectively ( Table 6).

**Table 6.** Sensitivity and specificity of techniques compared with formal ether.

Technique		Formal ether concentration technique		Total	Sensitivity	specificity
		+ve	-ve		50%	100%
Wet preparation technique	+ve	24	0	24		
	-ve	24	86	110		
Total		48	86	134		
Saturated sugar floatation technique	+ve	22	0	22	45%	100%
	-ve	26	86	112		
Total		48	86	134		

## Discussion

It was evident from the results that the overall prevalence of gastrointestinal parasites among children's schools (Saeed primery boys school, Ebaid Khatiem primery boys school, the martyr Abdulsalam primary girls school and Ebaid Khatiem primery girls school), in Al-kalakla was high (35.8%) and was found to be higher than the rate reported by Muhajir *et al*. (2017) in Alkalakal (30%) ^14^. Nevertheless, our rate was found to be lower than that reported by Gabbad and Elawad (2014) in Elengaz, Sudan (64.4%) ^15^. The study found that females had a slightly higher occurrence of gastrointestinal parasites (38.8%) than males (32.8%). This finding was not aligned with Muhajir *et al*. (2017) in Al-kalakla, which found higher rates of infection in males (16.5%) compared to females (13.5%) ^14^.

The highest prevalence rate (53.8%) among the 12–14 age group was reported in this study. This rate did not agree with that of Magambo *et al*. in Southern Sudan, who reported the most affected age group was those 6–10 years ^16^. The higher occurrence of gastrointestinal parasite may be due to student’s hygiene and lack of adequate water cycle in these schools.

The findings of this study indicated that the common gastrointestinal parasites in children's schools were *E. histolytica* (16.4%), *H. nana* (10.4%), *E. coli* (7.5%), *A. lumbricoids* (5.2%), *S. mansoni* (5.2%), *G. Lamblia* (3.7%) and *Taenia* spp. (1.5%), while Muhajir *et al*. reported *E. histolytica* the most common gastrointestinal parasites in Al-kalakla (15.5%), *G. lamblia* (12.5%), *H. nana* (1.5%) and *S. mansoni* (0.5%) ^14^. However, Gabbad and Elawad in Elengaz, Sudan, revealed that *G. lamblia* was the predominant gastrointestinal parasite (33.4%), followed by *H. nana* (26.4%), *T. saginata* (8.6%), *Enterobius vermicularis* (6.2%), *S. mansoni* (4.4%) and *E. histolytics* (3.6 %) ^15^.

As far as the detection levels for the three methods used were concerned, it was evident that the best detection rate (35.8%) was recorded for the formal ether concentration method and the lowest rate (16.4%) was recorded for the saturated sugar flotation method, while the wet preparation method showed a detection rate of 17.9%. Our results on formal ether were not in agreement with Eisa in Keryab Village, Sudan, who reported a 90% detection rate ^17^. However, the detection rate reported in our study was lower than the detection rate reported by Eman (44%) ^18^.

The study found that the detection rate for wet preparation (17.9%) was lower than the detection rate reported by Eman (41.4%) in Southern Sudan ^18^. Furthermore, detection rate for saturated sugar flotation technique (16.4%) was lower than the detection rate for Duria in Khartoum, Sudan, reported (58.6%) ^18^.

Surprisingly, the findings showed a sensitivity of 50% and a high specificity (100%) of the wet preparation method and a sensitivity of 45% and a high specificity (100%) of the saturated sugar flotation method. This could probably be attributed to severe intestinal parasite infections among subjects studied in this study.

## Conclusion

From the outcomes, it can be concluded that gastrointestinal parasites are common among children's schools in Al-kalakla, Khartoum state. Furthermore, prevalence rate was significantly greater among females, the highest rates of infection were recorded in the 12–14-years age group and, lastly, the formal ether concentration method showed the highest sensitivity level for the identification of distinct gastrointestinal parasites.

## Data availability

### Underlying data

Figshare: Hala and Ahmed file.sav. https://doi.org/10.6084/m9.figshare.9804869.v1 ^13^.

This project contains the following underlying data:

- Hala and Ahmed file.sav (parasites detected in stool samples for each child, with method used to detect them)
- Data dictionary (2).docx

Data are available under the terms of the Creative Commons Zero “No rights reserved” data waiver (CC0 1.0 Public domain dedication).
