# Supplementary material for: Frequencies of gastrointestinal parasites among students of primary school in Al Kalakla Locality, Khartoum State, Sudan: a cross-sectional study
Source: F1000Res. 2020 Sep 7;8:1719. Originally published 2019 Oct 4. [Version 3] doi: 10.12688/f1000research.20610.3 (PMC7459890; doi:10.12688/f1000research.20610.3)
Supplement: Peer review history

# Peer review: Reviewer response for version 2

**DOI:** 10.5256/f1000research.23392.r66823

**Published:** 2020-08-28

**Authors:** Sandrine Lydie Oyegue-Liabagui

**Affiliations:** 1 Ecole Doctorale d’Afrique Centrale en Infectiologie Tropicale de Franceville (ECODRAC), Université des Sciences et Techniques de Masuku, Franceville, Gabon; 2 Unité d’Evolution, Epidémiologie et Résistances Parasitaires (UNEEREP), Centre International de Recherches Médicales de Franceville CIRMF), Franceville, Gabon

**Background**

 The study evaluates the frequencies of the intestinal parasite among primary school students.

 The authors do not clearly present the objective of their work. Rather, they have the best techniques for identifying more parasites. The question then arises whether the aim of this work is the evaluation of parasite identification techniques or the determination of the prevalence of intestinal parasites among children of school age.

 Therefore that the authors clearly indicate the objective of the study.

**Materials and Methods**

- Study background: This paragraph describes the type, location, and period of study. It might be more suitable to name it “Study area and Design”.
- Direct smear examination: in this paragraph, please indicate when the sample is considered negative.
- Identification of parasites: This paragraph "Identification of parasites" should be included in the description of the methods used. e.g.: this method is used to detect eggs, cysts, vegetative forms, etc.

 

**Results: **

- Overall occurrence of intestinal parasites: replace “occurrance” by occurrence.
- It would be more interesting to correctly describe the prevalence of each intestinal parasite. Which parasite is most prevalent in the general population?
- Occurrence of intestinal parasite by variables: By age group what types of parasites are found? As far as the analysis of prevalence by age group is concerned, it would also have been interesting to make a pair-wise comparison, analysing the groups two by two using the Mann-Whitney test.
  The authors only indicate that there were 20 people infected with more than one parasite. However, they do not make any mention of the different polyinfections observed in the infected study participants. Therefore, a more detailed analysis of the observed polyinfections would be necessary.

 

**Discussion:**

- There is not much discussion about the distribution of parasites by age group and why the 12-14 year old age group is the most infected as opposed to the youngest, who seem to be the least attentive to hygiene measures **.**

Is the work clearly and accurately presented and does it cite the current literature?

Yes

If applicable, is the statistical analysis and its interpretation appropriate?

Yes

Are all the source data underlying the results available to ensure full reproducibility?

Yes

Is the study design appropriate and is the work technically sound?

Yes

Are the conclusions drawn adequately supported by the results?

Yes

Are sufficient details of methods and analysis provided to allow replication by others?

Yes

Reviewer Expertise:

NA

I confirm that I have read this submission and believe that I have an appropriate level of expertise to confirm that it is of an acceptable scientific standard.

# Peer review: Reviewer response for version 2

**DOI:** 10.5256/f1000research.23392.r57060

**Published:** 2019-12-02

**Authors:** Hari Prasad Nepal, Rama Paudel

**Affiliations:** 1 Trinity Medical Sciences University, Kingstown, Saint Vincent and the Grenadines; 2 Department of Microbiology, School of Medicine, Trinity Medical Sciences University, Kingstown, Saint Vincent and the Grenadines

The previous comments have been addressed in the second version of the manuscript.

Is the work clearly and accurately presented and does it cite the current literature?

Partly

If applicable, is the statistical analysis and its interpretation appropriate?

Partly

Are all the source data underlying the results available to ensure full reproducibility?

Partly

Is the study design appropriate and is the work technically sound?

Partly

Are the conclusions drawn adequately supported by the results?

Partly

Are sufficient details of methods and analysis provided to allow replication by others?

Partly

Reviewer Expertise:

Infectious diseases (including parasitic diseases), antimicrobial resistance, and medical education

We confirm that we have read this submission and believe that we have an appropriate level of expertise to confirm that it is of an acceptable scientific standard.

# Peer review: Reviewer response for version 1

**DOI:** 10.5256/f1000research.22663.r55524

**Published:** 2019-10-23

**Authors:** Hari Prasad Nepal, Rama Paudel

**Affiliations:** 1 Trinity Medical Sciences University, Kingstown, Saint Vincent and the Grenadines; 2 Department of Microbiology, School of Medicine, Trinity Medical Sciences University, Kingstown, Saint Vincent and the Grenadines

The study provides basic information about the frequencies of gastrointestinal parasites among children of a school in Sudan. The findings of this study may be useful to people of public health interest in the local region but can not be generalised. The following observations were noted while reviewing this manuscript:

**Title:**

1. Since the study:
  a. detects only the intestinal parasites,
  b. does not reveal anything about the clinical symptoms of parasitic infections and
  c. involves children of only one school, the title should be changed to 'Frequencies of gastrointestinal parasites among students of a primary school in Al Kalakla Locality, Khartoum State, Sudan: a cross-sectional study'.

**Methods:**

1. **Study population**: The authors should clearly mention how the sampling was determined and how each of the participants was selected (e.g, random sampling).
2. **Identification of parasites**: The information  (whole paragraph) about identification of parasites should be mentioned/moved after the last concentration method (i.e just before data analysis).
3. **Saturated sugar flotation technique:** The first and second sentences can be joined with removal of full stop after first sentence and use of 'and' 3rd line: 'sample of fecal' should be 'fecal sample'.
4. **Data analysis**: specificity should be determined by multiplication of the fraction by **100 (not by 10).**
5. The authors should clearly mention (under 'Methods') whether all the samples were subject to examination by all the three methods in the study. 

**Results**

1. The term **'occurrence'** would be better than 'prevalence' (which has its specific epidemiological meaning) in the title/contents of first two paragraphs.
2. **3rd line of 2nd paragraph**: **'years**' should be added after the age groups mentioned (i.e, 6–8, 9–11 and 12–14 **years). **

**Discussion**

1. **First sentence: **The study done at one school can not be generalised. **This sentence can be restructured as '**It was evident from the results that the overall occurrence of gastrointestinal parasites among children of a school *(school name can be mentioned*) in Al-kalakla was high........ 
2. The study needs to discuss the reasons for higher occurrence: 
  a. of the parasites in this study compared to that of other studies,
  b.of the parasites in females compared in males
  c. of the parasites in 12–14 years of age group compared in other age groups. 

**Conclusion:**

 The study done at one school can not be generalised. The authors can note the first comment made under 'discussion' and conclude accordingly.

Is the work clearly and accurately presented and does it cite the current literature?

Partly

If applicable, is the statistical analysis and its interpretation appropriate?

Partly

Are all the source data underlying the results available to ensure full reproducibility?

Partly

Is the study design appropriate and is the work technically sound?

Partly

Are the conclusions drawn adequately supported by the results?

Partly

Are sufficient details of methods and analysis provided to allow replication by others?

Partly

Reviewer Expertise:

Infectious diseases (including parasitic diseases), antimicrobial resistance, and medical education

We confirm that we have read this submission and believe that we have an appropriate level of expertise to confirm that it is of an acceptable scientific standard, however we have significant reservations, as outlined above.
